# Efficient coralline algal *psb*A mini barcoding and High Resolution Melt (HRM) analysis using a simple custom DNA preparation

**DOI:** 10.1038/s41598-018-36998-6

**Published:** 2019-01-24

**Authors:** Marc B. Anglès d’Auriac, Line Le Gall, Viviana Peña, Jason M. Hall-Spencer, Robert S. Steneck, Stein Fredriksen, Janne Gitmark, Hartvig Christie, Vivian Husa, Ellen Sofie Grefsrud, Eli Rinde

**Affiliations:** 1 Norwegian Institute for Water Research (NIVA), N-0349 Oslo, Norway; 2 Institut Systématique Evolution Biodiversité (ISYEB), Muséum national d’Histoire naturelle, CNRS, Sorbonne Université, EPHE, 57 rue Cuvier, CP 39, 75005 Paris, France; 3 BIOCOST Research Group & CICA, Universidade da Coruña, Campus de A Coruña, 15071, A Coruña, Spain; 4 School of Marine and Biological Sciences, Plymouth University, Plymouth, UK; 5 Shimoda Marine Research Centre, Tsukuba University, Tsukuba, Japan; 6 University of Maine, School of Marine Sciences, Orono, USA; 7 University of Oslo, Oslo, Norway; 8 Institute of Marine Research (IMR), Bergen, Norway

## Abstract

Coralline algae form extensive maerl and rhodolith habitats that support a rich biodiversity. Calcium carbonate harvesting as well as trawling activities threatens this ecosystem. Eleven species were recorded so far as maerl-forming in NE Atlantic, but identification based on morphological characters is unreliable. As for most red algae, we now use molecular characters to resolve identification of these taxa. However, obtaining DNA sequences requires time and resource demanding methods. The purpose of our study was to improve methods for achieving simple DNA extraction, amplification, sequencing and sequence analysis to allow robust identification of maerl species and other coralline algae. Our novel and easy DNA preparation method for coralline algae was of sufficient quality for qPCR amplification and sequencing of all 47 tested samples. The new *psb*A qPCR assay successfully amplified a 350 bp fragment identifying six species and uncovering two new Operational Taxonomic Units. Molecular results were corroborated with anatomical examination using i.e. scanning electron microscopy. Finally, the qPCR assay was coupled with High Resolution Melt analysis that successfully differentiated the closely related species *Lithothamnion erinaceum* and *L*. cf. *glaciale*. This DNA preparation and qPCR technique should vitalize coralline research by reducing time and cost associated with molecular systematics.

## Introduction

Coralline algae (Rhodophyta with calcareous cell walls belonging to the orders Corallinales, Hapalidiales and Sporolithales) can live unattached on the seabed to form maerl or rhodolith deposits (*sensu* Irvine and Chamberlain 1994). They grow slowly in northern Europe (maerl branches growth rate are about 1 mm year^−1^) where they form diverse biogenic habitats^1,2^. Maerl beds are analogous to coral reefs, seagrass meadows and kelp forests in being structurally and functionally complex perennial habitats that support a very rich biodiversity^3–5^. In the NE Atlantic, the three-dimensional structure of maerl beds are habitats for more than 500 animal taxa and 300 seaweed taxa that live among or attached to the maerl as well as on shells and pebbles/stones found interspersed^6^. In the European literature, the term maerl is applied to branched unattached coralline algae that are often composed by one, or occasionally/rarely a few species^7^. This contrasts with other non-geniculate corallines that encrust an inorganic core such as shells or stones and thus can include several taxa, sometimes overgrowing each other^7^. Maerl beds have a patchy distribution, and in each region they are composed of different coralline species^6,8,9^ reflecting their biogeography.

Coralline algae are perhaps the most abundant and widespread organism to occupy hard substratum within the photic zone. They are found on all coasts from the tropics to the poles. Their calcium carbonate thallus allows them to colonise habitats with high hydrodynamism contributing to shaping the seascape^10^. With more than 600 species currently recognized^11^, the subclass Corallinophycidae is one of the most diverse groups of red algae. Coralline algae show high morphological variation within a single taxon (e.g.^12^), and convergences among phylogenetically distant taxa^13^. These characteristics make their identification based on morphological and anatomical characters alone difficult. Coralline algae along the Norwegian coast were largely sampled and studied in mid-1800^14–18^; therefore, a re-evaluation of coralline diversity using molecular characters is needed. Recent works have re-assessed coralline species diversity in NE Atlantic^8,9^, although such work is scarce in Norway.

In recent years, assessments of species diversity have benefited from the development of molecular tools, and they have been successfully applied to identify coralline species and cryptic taxa^8,9,19–26^. However, currently available sequence data for coralline species are far from being close to a comprehensive representation of the actual diversity^8^. Furthermore, DNA preparation for calcareous algal species typically involves time and cost consuming steps such as careful removal of a clean fragment with a razor blade or similar equipment, grinding followed with DNA extraction and purification. Hence, more cost effective DNA preparation methods as well as easier DNA analysing methods, have the potential to speed up the progress of molecular work on corallines, and enhance the completeness of a public data base for this complex algal group^27^.

Here, we develop and evaluate a cost effective and simple new approach of DNA preparation, PCR amplification, High Resolution Melt (HRM) analysis, and DNA sequencing for coralline species identification. To our knowledge, it is the first time that HRM analysis, a cheap and fast alternative to sequencing, is applied to marine macroalgae for diagnostic purposes, increasing the toolbox available to marine biologists. Our case study, which focused on Norwegian maerl beds, enables us to present preliminary results on coralline species diversity forming maerl beds from this region.

## Results

**Figure 1:**
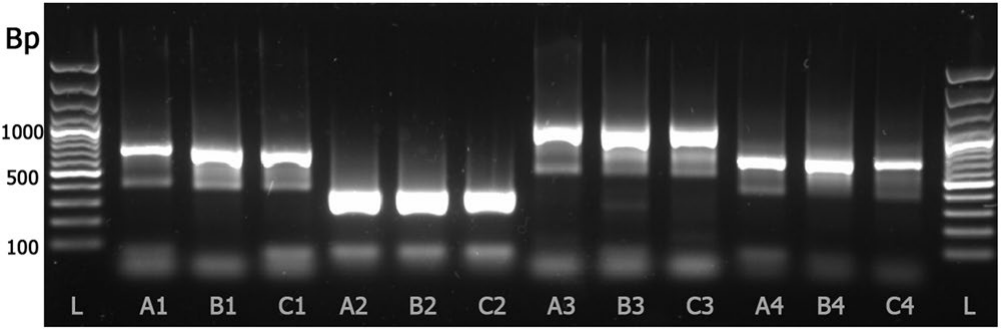
DNA preparation (3 methods) and PCR (4 protocols) comparisons. 1 Kb DNA ladder (L). Grinded sample with QuickExtract followed with Monarch purification, eluate diluted 10^−1^ in Di H_2_O (A1, A2, A3 & A4). Whole sample with QuickExtract, eluate diluted 10^−2^ in Di H_2_O (B1, B2, B3 & B4). Whole sample with QuickExtract followed with Monarch purification, eluate diluted 10^−1^ in Di H_2_O (C1, C2, C3 & C4). Primers GazF1 & GazR1 producing a 610 bp amplicon (A1, B1 & C1). Primers psbA21-350F & psbA22-350R producing a 350 bp amplicon (A2, B2 & C2). Primers psbA-F1 & psbA-R1 producing a 957 bp amplicon (A3, B3 & C3). Primers psbA-F1 & psbA-R600 producing a 600 bp amplicon (A4, B4 & C4). The full-length gel is presented in Supplementary Figure S3.

The new and simple DNA sample preparation “B” (Fig. 1, lanes B1, B2, B3 & B4) was compared with two other methods involving sample grinding “A” and DNA purification “C” (Fig. 1 lanes A1, A2, A3, A4 & C1, C2, C3, C4). In addition to the new primer pair psbA21-350F & psbA22-350R (Fig. 1 lanes A2, B2 & C2), three additional primer pairs amplifying larger products from the psbA and mitochondrial COI genes (Table 1) were used for evaluation of the DNA preparation method (Fig. 1, lanes A1, B1, C1, A3, B3, C3, A4, B4 and C4). All three tested DNA preparation methods were successful in amplifying products with the 4 PCR protocols yielding products varying from 350 to 957 bp (Table 1). However, the new primer pair showed the strongest product amplification with all three DNA preparation protocols (Fig. 1 lanes A2, B2 and C2) and all four primer pairs showed best amplification with the new simple DNA sample preparation method (Fig. 1 lanes B1, B2, B3 and B4). The new primers and DNA preparation were used for analysing all 47 coralline individuals.

**Table 1:** Primers.

Target	Primer	Sequence 5′-3′	Tm °C	Prod. (bp)	Reference
Plastid gene encoding the D1 protein of photosystem II (*psb*A)	psbA-F1	ATGACTGCTACTTTAGAAAGACG	57.1		^31, 46– 48^
	psbA600R	CCAAATACACCAGCAACACC	57.3	600	
	psbA-R1	GCTAAATCTARWGGGAAGTTGTG	58	957	
	psbA21-350F	TTCTCTGATGGAATGCCTCTA	55.2		This study
	psbA22-350R	CAGATACCTACTACAGGCCATA	56	350	
Mitochondrial COI-5P	GazF1	TCAACAAATCATAAAGATATTGG	51.7		^31, 49^
	GazR1	ACTTCTGGATGTCCAAAAAAYCA	56.2	710	

**Figure 2:**
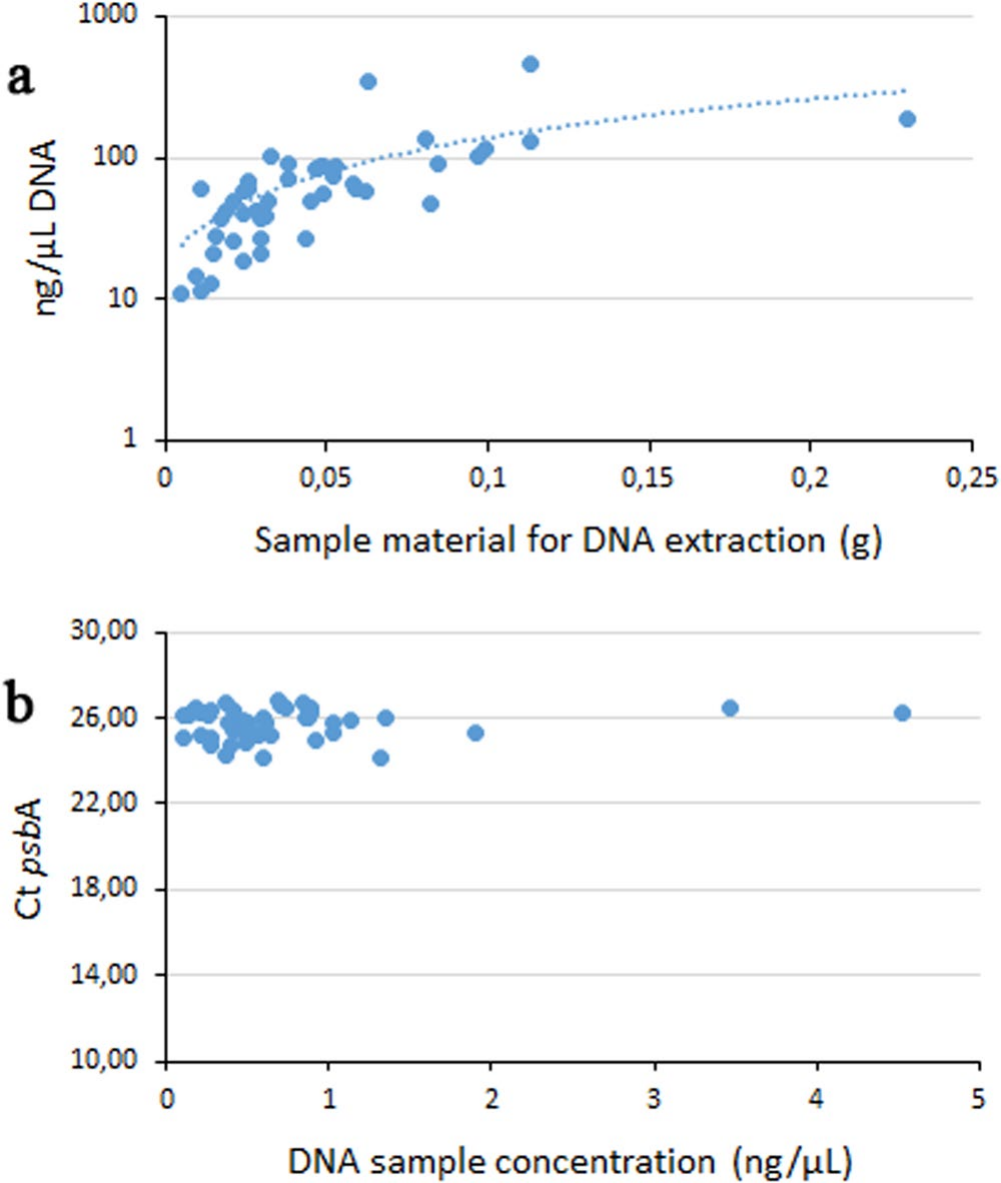
Sample DNA preparation evaluation. (**a**) DNA concentration related to sample quantity. (**b**) qPCR Ct result as function of DNA concentration (all samples diluted 100 time in DI H_2_O).

**Figure 3:**
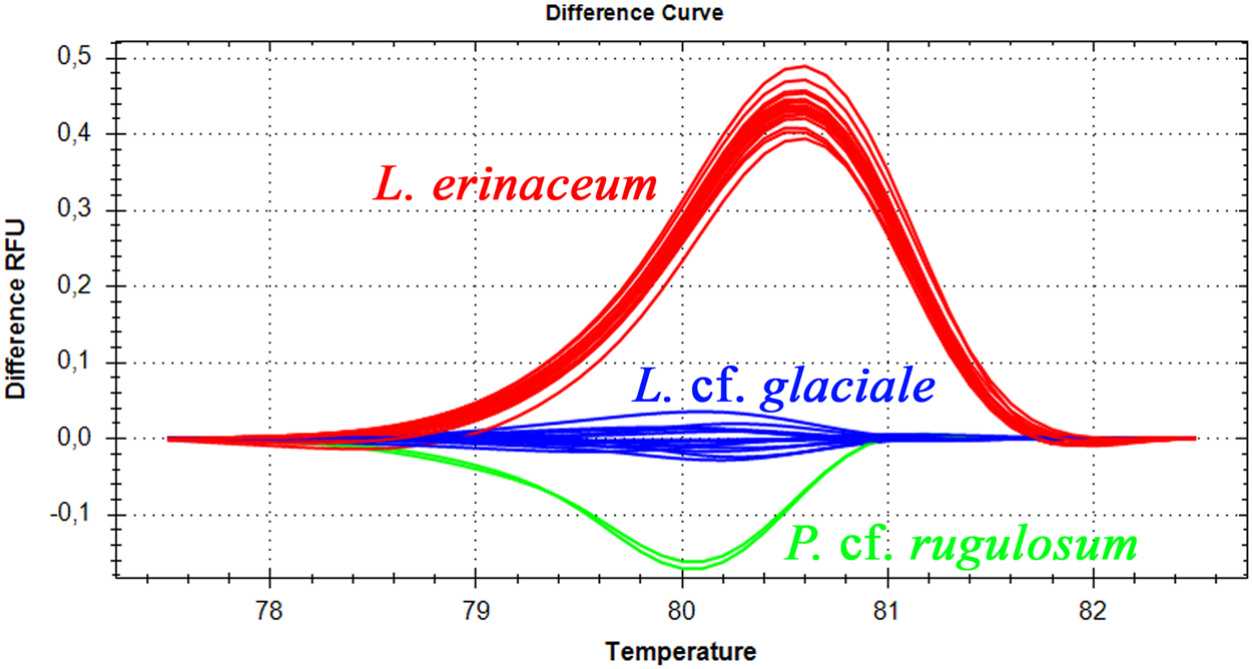
High Resolution Melt analysis of psbA 350 bp product. The central blue cluster shows all analyzed *L*. *glaciale* and was used as the reference cluster.

**Figure 4:**
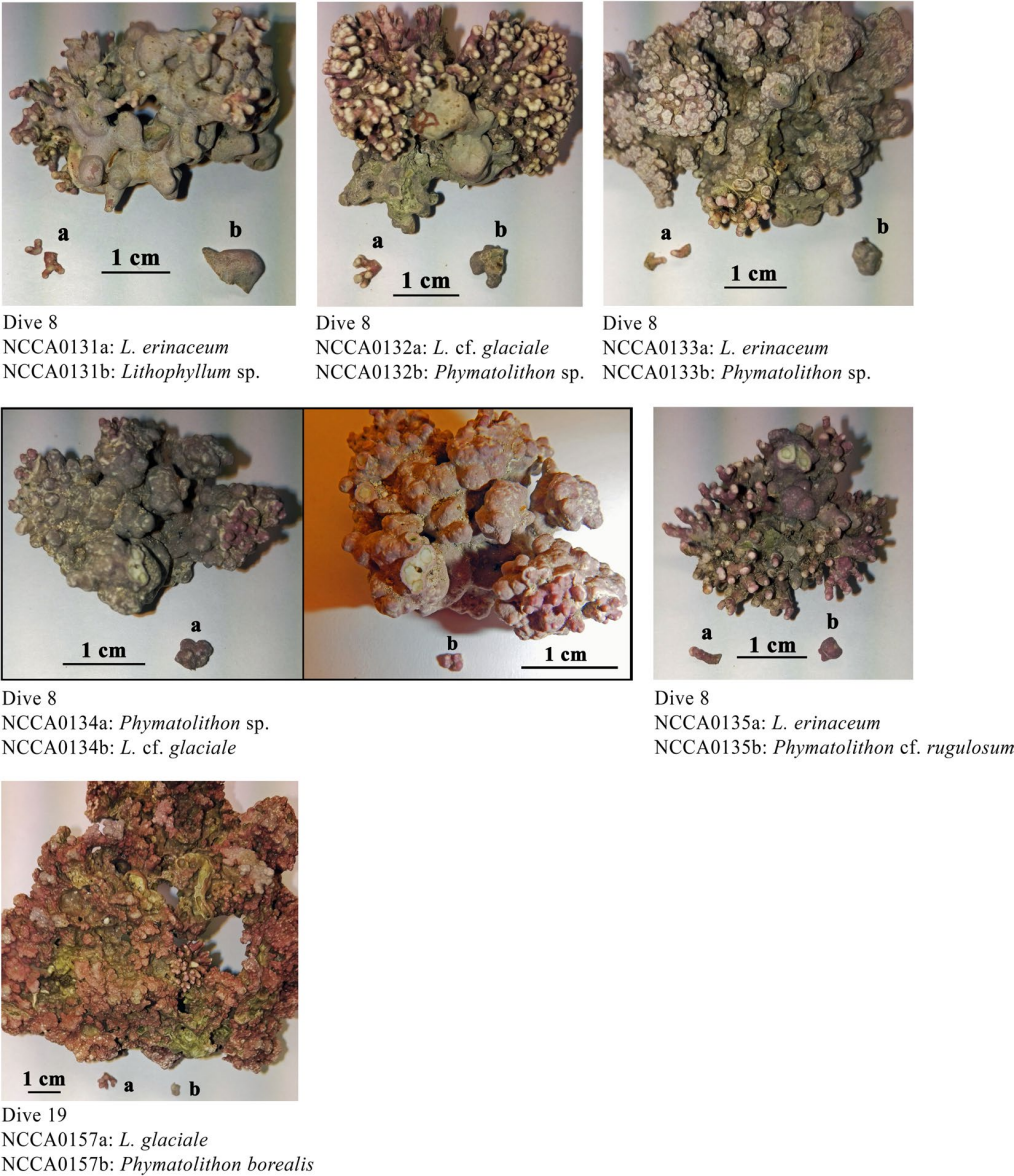
Six two-species maerl specimens. Specimen NCCA0131, NCCA0132, NCCA0133, NCCA0134 & NCCA0135 from d8-Krøttøya and NCCA0157 from d19-Sørøya.

The aliquots collected for the simple DNA sample preparation (0.005 to 0.023 g) yielded DNA concentrations varying from 11 to 452 ng/µL (Fig. 2a). Although there was a positive correlation between material input and DNA concentration yield, all samples diluted 100 X in diH2O produced a Ct at 25.7 + /− 0.7 (Figs 2b and S1a). A single mastermix for real time PCR was used for product assessment by melt curve (Supplementary Fig. S1b), HRM analysis (Fig. 3), and was further diluted as template for cycle sequencing. The produced sequences and sample information are available at BOLD (the Barcode of Life Data Systems http://www.boldsystems.org/) with accession numbers MG191372-MG191375, MG191658-MG191703 and MH034113. Some coralline algal specimens presented more than one distinct morphology. To reveal the possible presence of several species, separate aliquots were sampled from these. Six specimens were suspected of being composed of several taxa (five from d8-Krøttøya & one from d19-Sørøya). All six specimens revealed the occurrence of two different species as shown in Fig. 4. The taxonomic results are presented in Table 2 in a matrix format showing all eight taxa and their occurrence either as a single maerl species or co-occurrence with another species. The two most common maerl species were *Lithothamnion* cf. *glaciale* and *L*. *erinaceum*, with 21 and 10 specimens respectively, which together constituted 66% of the samples. We use *L*. cf. *glaciale* because molecular data from the lectotype of *L*. *glaciale* are not currently available for comparison. *Phymatolithon calcareum* which is considered to be a major maerl-forming species in Europe appeared to be scarce (three specimens, from Karmøy in Southern Norway, d21). Both dominant maerl species, *Lithothamnion erinaceum* and *Lithothamnion* cf. *glaciale*, were found alone for 86 and 70% of the collections respectively, the remainder growing together with other coralline species (for three specimens each, See Fig. 4). These six multispecies specimens were found at Krøttøya d8 (n = 5) and Sørøya d19 (n = 1) and the associated coralline species were *Phymatolithon borealis* (n = 1), *Phymatolithon* cf. *rugulosum* (n = 1) and two new OTUs, *Phymatolithon* sp. (n = 3), and *Lithophyllum* sp. (n = 1).

**Table 2:** Taxa sampled in 2016 along the Norwegian coast, and co-occurrence matrix for species found together.

Species	*L. cf. glaciale*	*L. erinaceum*	*P. borealis*	*P. calcareum*	*P. cf. rugulosum*	*Phymatolithon* sp.	*L. laeve*	*Lithophyllum* sp.	Tot.
*Lithothamnion* cf. *glaciale*	18*	0	1**	0	0	2**	0	0	**21**
*Lithothamnion erinaceum*	0	7*	0	0	1**	1**	0	1**	**10**
*Phymatolithon borealis*	1**	0	5*	0	0	0	0	0	**6**
*Phymatolithon calcareum*	0	0	0	3*	0	0	0	0	**3**
*Phymatolithon* cf. *rugulosum*	0	1**	0	0	1*	0	0	0	**2**
*Phymatolithon sp*.	2**	1**	0	0	0	0	0	0	**3**
*Leptophytum laeve*	0	0	0	0	0	0	1*	0	**1**
*Lithophyllum* sp.	0	1**	0	0	0	0	0	0	**1**
Total	**21**	**10**	**6**	**3**	**2**	**3**	**1**	**1**	**47**

*Single species maerl or rhodolith sample; **co-occurrence of two species on the same maerl specimen, found in dive 8 (5) & dive 19 (1).

Anatomical examination of sequenced specimen corroborated our molecular results (See Supplementary Fig. S2). Thus, the most abundant species identified as *Lithothamnion* cf. *glaciale* and *L*. *erinaceum* showed features described for the genus *Lithothamnion* such as cell fusions as the only type of connections between cells of contiguous filaments, the occurrence of flared epithallial cells and subepithallial cells as long or longer than cells subtending them in vertical section, and multiporate sporangial conceptacles (Supplementary Fig. S2A–F). Species belonging to the genus *Phymatolithon* shared with *Lithothamnion* the presence of cell fusions and multiporate sporangial conceptacles, but they are characterized by producing domed epithallial cells, and subepithallial cells usually shorter than cells subtending them in vertical section (Supplementary Fig. S2G–K). By contrast the genus *Lithophyllum* has exclusively secondary pit-connections between cells of contiguous filaments (Supplementary Fig. S2L,M).

**Figure 5:**
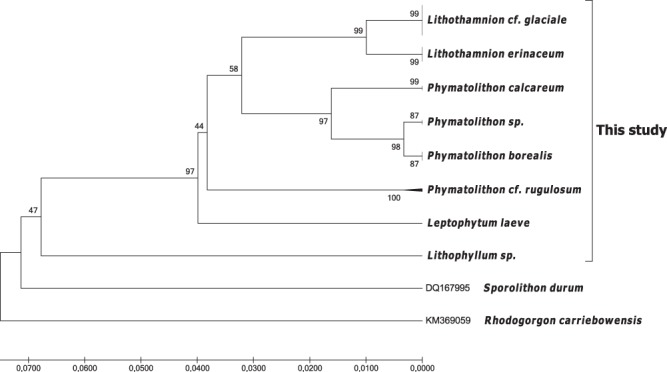
Species delimitation among sequenced specimens. Tree inferred by unweighted pair group means analysis tree for the psbA sequences using Kimura 2-parameter substitution model. The percentage of replicate trees in which the associated taxa clustered together in the bootstrap test (10,000 replicates) is shown next to the branches. In addition to the 47 coralline specimens sequenced for this study, 2 sequences, DQ167995 & KM369059, were used to serve as outgroup. GenBank accession numbers for the sequences generated for this study are indicated in Additional Table 2.

HRM analysis produced unambiguous distinction between *Lithothamnion erinaceum*, *Lithothamnion* cf. *glaciale* and *Phymatolithon* cf. *rugulosum* (Fig. 3). The other species did not cluster adequately for differentiation using HRM. Hence the method may be used to easily differentiate the two closely related species *L*. *erinaceum* and *L*. cf. *glaciale* without requiring sequencing. Sequencing of the 350 bp product was used to infer taxonomic relationship between the individuals (Fig. 5). It shows the close relationship between *Lithothamnion* cf. *glaciale* and *L*. *erinaceum* as well as the clustering of *Phymatolithon* sp. with *Phymatolithon borealis* and *P*. *calcareum*.

The distribution of the eight coralline species found on maerl beds along the Norwegian coast is indicated in Supplementary Tables S1 and S2, showing, amongst other, that *P*. *calcareum* was only found in the south at Karmøy.

## Discussion

Molecular tools are steadily becoming more important for taxonomic work, and the development of simpler procedures and a reduction in costs will be important to widen the use of these techniques. DNA preparation, often the first step in a molecular laboratory, can be time-consuming and expensive. Simple DNA preparation methods without purification steps have been developed for marine organisms such as mussels^28^ sea urchins^29^ and oysters^30^. Typical DNA preparation for coralline algae involves grinding or pulverization in liquid nitrogen, followed by extraction using commercially available kits^31,32^. This approach is time-consuming, may increase cross contamination risk with the production of sample powder, and is costly due to the price of extraction kits. The method we propose simplifies and speeds up the required work, although good knowledge of coralline algae and their biology is still required to ensure appropriate tissue selection for DNA preparation. A small aliquot of the sample is separated from the studied coralline (see Fig. 4) and directly added to a 1,5 mL Eppendorf tube to which qPCR compatible lysis buffer is added prior to incubation. The method is robust as aliquots are between 0.005 and 0.23 g and yet produced homogenous qPCR results after diluting the DNA preparation 100 x in diH_2_O (see Figs 2 and S1). Further, a common difficulty associated with barcode sequencing is degraded DNA, in particular when samples originate from old collections preserved in museums, or suffer from poor storage conditions, resulting in low amplification rates. This problem has been addressed by developing new primers to amplify a shorter target than those in reference assays (see Table 1). Reducing the size of the targeted amplicon by developing such mini barcodes^33^ increases amplification rates and yet retains sufficient information for taxonomic purposes^34^. The new primers used in this study target a 350 bp section of the *psb*A gene, retaining sufficient variability for differentiating the closely related species *Lithothamnion* cf. *glaciale* from *L*. *erinaceum* with six Single Nucleotide Polymorphisms (SNPs). Sequence variation among PCR products of same length may be easily differentiated, without sequencing, by using HRM for various diagnostic purposes such as for example fish^35,36^, mollusc^37,38^ and microalgae^39^ species and genotypic sex identification. In this study HRM was used on the 350 bp *psb*A amplicons to identify three maerl species, *Lithothamnion erinaceum*, *Lithothamnion* cf. *glaciale* and *Phymatolithon calcareum*, from the Norwegian coasts. HRM is a very cheap diagnostic alternative to sequencing for identification purposes. Although sequencing has become cheaper when outsourced one still must prepare, send the samples for sequencing and await results. Within the scope of this study, HRM enables unambiguous differentiation between *Lithothamnion* cf. *glaciale* and *L*. *erinaceum*, the two most common species found in the studied area, which are also morphologically close and difficult to differentiate from each other. While the two *Lithothamnion* species were abundant, and are common in circumpolar regions (Iceland, Scotland, British Columbia^8,9^), *Phymatolithon calcareum* was scarce in Norwegian maerl beds. This latter species is, however, reported as one of the major maerl species in Europe along with the temperate *Lithothamnion corallioides*, and both are listed in the annex V of the EU Habitats Directive. Apart from these three maerl species detected in Norway, several other coralline taxa were found growing together with *L*. *glaciale* or *L*. *erinaceum* or formed rhodoliths themselves around stones, shell fragments or the remains of other coralline algae, *viz*. *Phymatolithon borealis*, *Phymatolithon* cf. *rugulosum*, *Phymatolithon* sp., *Lithophyllum* sp. and *Leptophytum laeve*.

Meta barcoding is an alternative method to identify species in a bulk sample. Even if it is extremely efficient, the method developed in this study presents the advantage over metabarcoding that it allows traceability between the algal specimen and the sequence, a necessary step in building up a DNA library of life. In some cases, multiple coralline species occurred together in the same unattached maerl specimen; this method was able to detect and identify these associations between coralline species, that mainly corresponded to the crustose taxa *Phymatolithon borealis*, *P*. cf. *rugulosum*, *Phymatolithon* sp. and *Lithophyllum* sp. associated to either *Lithothamnion* cf. *glaciale* or *L*. *erinaceum* (see Table 2 and Fig. 4). The anatomical examination of the specimens confirmed that molecular results obtained matched the diagnostic features described for these coralline taxa. These tools will help unravel species determination of coralline algae shedding light on the way they may interact between them and with their environment. Knowledge on how these organisms contribute to forming the seascape they belong to should provide new insights for coastal management and in particular restoration.

## Conclusions

Three different DNA preparation methods, as well as four PCR protocols targeting *psb*A and COI-5P, were evaluated on coralline algae sampled from Norwegian maerl beds. The results show that the new simple DNA preparation method produced the best results, as well as the shorter *psb*A PCR which produced amplicons for all 47 studied samples with sufficient sequences variability for taxa identification and for successful HRM analysis of the two most commonly occurring maerl taxa in northern Europe, *L*. *erinaceum* and *L*. cf. *glaciale*. Finally, a single qPCR mastermix was used for both product assessment, HRM and cycle sequencing avoiding the need for running gels and performing a second PCR amplification with a different mastermix for sequencing. These new simple and low-cost methods should ease the acquisition of DNA sequences needed to foster investigation on coralline diversity with molecular systematics tools.

## Methods

### Sampling and DNA preparation

A total of 47 free-living coralline specimens (maerl and rhodoliths) were collected and analysed from five areas along the Norwegian coast in 2016 and 2017 (Supplementary Table S1). Each sample was labelled –NCCA- with a unique 4-digit identifier. When a sample appeared to be composed of several species, an additional letter (“a”, “b”, etc.), was added to the corresponding NCCA number. Pictures of all samples and aliquots used for DNA preparation can be found on Barcode of Life Data Systems (BOLD)^40^ Dataset - DS-NOCCAMET Corallines DNA preparation & psbA HRM, associated to the produced sequences (Supplementary Table S2). The samples were conserved by drying in heating cabinets at 60 °C, overnight.

We developed a simple DNA preparation protocol without any purification steps. Each sample was first thoroughly brushed using a common dish brush with tap water and rinsed with deionized water. A small aliquot was broken off from the main sample, weighting between 0.005 and 0.23 g (Figs 2A and 4) and added to a 1.5 mL Eppendorf tube without any further preparation. QuickExtract (Epicentre Technologies Corporation, Madison, USA) buffer was added, 200 µL per tube, followed with an incubation at 65 °C for 15 min and 98 °C inactivation for 5 min. DNA concentration and quality was measured using NanoDrop ND-1000 spectrophotometer (www.nanodrop.com). The lysates were further diluted 10^−2^ in DI water for PCR amplification followed with cycle sequencing. For method evaluation only, grinding was also performed using a rotary tool (Dexter 135 W), as well as DNA purification using Monarch PCR & DNA Cleanup Kit (New England Biolabs Inc.). A single non-identified coralline algal sample was used for the method evaluation. All 47 samples (Supplementary Table S2) were analysed using the new method.

### Primer design, PCR & HRM

Oligo7 v7.60^41^ was used for designing primers psbA21-350F and psbA22-350R for amplification of a 350 bp fragment of the plastid encoded *psb*A gene. Three additional primer pairs targeting either the *psb*A or mitochondrial COI genes were used for method development. Primer sequences are given in Table 1. PCR amplifications were performed using a CFX96 thermocycler (Bio-Rad, Hercules, CA, USA) in 15 μl reaction volume containing 7.5 μl SsoFast mastermix (Bio-Rad), 0.5 μM final concentration of each primer (Eurofins MWG, Ebersberg, Germany) and 1.5 μl sample. Reaction volume was completed with sterile deionised water. PCR optimal annealing temperature was determined by running a PCR with a temperature gradient. Optimized amplification for the newly designed primer pair psbA21-350F & psbA22-350R was carried out under the following conditions: a denaturing step for 2 min at 98 °C, followed by 40 cycles of 98 °C for 10 s, 57 °C for 20 s and 72 °C for 20 s. Melt curve analysis was performed between 65 °C and 95 °C using 0.2 °C increments with readings after 5 s. HRM analysis was performed using Precision Melt analysis software (Bio-Rad) by setting the difference curve analysis between 76.4 and 82.6 °C. SsoFast™ EvaGreen® mastermix was used for HRM analysis as well as for product input for sequencing. PCR product electrophoresis was done on a 1.7% agarose gel (Agarose I biotechnology grade, VWR) in 2X TAE buffer (VWR), run for 110 min at 70 V and visualized with GelRed (Biotium; www.biosciences.co.uk) staining using a Gel Doc™ XR + Documentation System (Bio-Rad) for acquiring pictures (Figs 1 and S3).

### Sequencing and genetic analysis

Cycle sequencing was performed in both directions using amplification primers and BigDye Terminator v3.1 kit (Life Technologies, Applied Biosystems). The PCR template was prepared by diluting PCR product 25X by adding 2 µl product to 48 µl DI water. Further, 1 μl of the prepared template was added to 0.5 μl Terminator mix, 0.32 μl 10 μM forward or reverse primer, 1.75 μl Terminator 5X buffer and 6.43 μl DI H_2_O. Cycle sequencing was done using an ABI 7500 qPCR machine as following: 96 °C for 1 min followed by 28 cycles of 96 °C for 10 s, 50 °C for 5 s and 60 °C for 3 min. Sequence purification was performed using BigDye XTerminator Purification kit (Life Technologies, Applied Biosystems) adding 10 μl XTermination solution and 45 μl Sam solution to each PCR sample well, reaching a final volume of 65 μl. The PCR plate was then sealed and vortexed for 30 min prior to being processed by an ABI3730XL DNA analyzer (Life Technologies, Applied Biosystems). Trace files analyses were performed using CodonCode Aligner v7 (CodonCode Corporation), sequence alignments were performed using MultAlin^42^ and the species delimitation was inferred using the UPGMA method (unweighted pair group means analysis) using MEGA version 7.0.21^43^. The genetic distances were computed using the Kimura 2-parameter method^44^ and are in the units of the number of base substitutions per site. The analysis involved 49 nucleotide sequences, including two reference sequences from GenBank. All positions containing gaps and missing data were eliminated. There were 307 positions in the final dataset.

### Anatomical studies of sequenced corallines

Consistent diagnostic features described for North Atlantic coralline genera (such as, type of intercellular connections between contiguous cell filaments, shape of epithallial, size of subepithallial initial cells, type of sporangial conceptacle –uniporate/multiporate-, thallus organization and construction; see Adey and Adey^45^ and Irvine and Chamberlain^7^) were observed using a scanning electron microscope (SEM, model JEOL JSM 6400, Universidade da Coruña, Spain). Before SEM examination, representative fragments for each sample sequenced were removed under a stereomicroscope and they were positioned on a stub and gold-coated to show surface view, transverse view and reproductive structures (if present).

All data generated or analysed during this study are included in this article (and its Supplementary Information files). DNA sequences are accessible at BOLD (the Barcode of Life Data Systems http://www.boldsystems.org/) and GenBank (https://www.ncbi.nlm.nih.gov/genbank/).

## Supplementary information

Dataset 1
