# Supplementary material for: Efficient coralline algal psbA mini barcoding and High Resolution Melt (HRM) analysis using a simple custom DNA preparation
Source: Sci Rep. 2019 Jan 24;9:578. doi: 10.1038/s41598-018-36998-6 (PMC6346035; doi:10.1038/s41598-018-36998-6)

Efficient coralline algal *psb*A mini barcoding and High Resolution Melt (HRM) analysis using a simple custom DNA preparation

Marc B. Anglès d'Auriac[1]*, Line Le Gall[2], Viviana Peña[3], Jason M. Hall-Spencer[4,5], Robert S. Steneck[6], Stein Fredriksen[7], Janne Gitmark[1], Hartvig Christie[1], Vivian Husa[8], Ellen Sofie Grefsrud[8] & Eli Rinde[1]

[1]Norwegian Institute for Water Research (NIVA), N-0349 Oslo, Norway. [2]Institut Systématique Evolution Biodiversité (ISYEB), Muséum national d'Histoire naturelle, CNRS, Sorbonne Université, EPHE, 57 rue Cuvier, CP 39, 75005 Paris, France. [3]BIOCOST Research Group & CICA, Universidade da Coruña, Campus de A Coruña, 15071, A Coruña, Spain. [4]School of Marine and Biological Sciences, Plymouth University, Plymouth, UK. [5]Shimoda Marine Research Centre, Tsukuba University, Tsukuba, Japan. [6]University of Maine, School of Marine Sciences, Orono, USA. [7]University of Oslo, Norway. [8]Institute of Marine Research (IMR), Bergen, Norway.

*Correspondence: mad@niva.no

Supplementary files

Table S1. Sample information; ecoregion, area, sample dive site, date, sample size, and geographic position (decimal degrees) of the sampling sites.

Ecoregion	County	Area	Dive site	Date sampled	Sample size	WGS84 DD Lat. / Long.
North Sea north	Troms	Krøttøya	d2A	25.07.2016	2	69.091095/ 16.502312
North Sea north	Troms	Krøttøya	d7	27.07.2016	1	69.075213 / 16.479483
North Sea north	Troms	Krøttøya	d8	27.07.2016	10*	69.073868/ 16.480023
Barents Sea	Finnmark	Porsangerfjorden	d9	01.08.2016	5	70.736207 / 25.658157
Barents Sea	Finnmark	Porsangerfjorden	d12	03.08.2017	5	70.369563 / 25.445291
Barents Sea	Finnmark	Porsangerfjorden	d15	04.08.2017	5	70.410093 / 25.337945
Barents Sea	Finnmark	Sørøya	d19	20.08.2016	9*	70.678488 / 23.225021
North Sea south/north	Nordland	Brønnøysund	d20	24.08.2016	6	65.467779 / 12.065683
North Sea south	Rogaland	Karmøy	d21	23.10.2016	4	59.3946189 / 5.204086

*Among which 10 were aliquoted from 2-species mærl plants (see Table S2)

Table S2. Sample species list with sequence access numbers.

Taxonomy	Dive site	Project ID	Bold ID	Acc. Nr.
Leptophytum laeve W.H. Adey	7	NCCA0141	NOCCA047-17	MG191684
Lithophyllum sp.	8	NCCA0131b	NOCCA038-17	MG191691<br>MG191374
Lithothamnion erinaceum Melbourne & J. Brodie	8	NCCA0131a	NOCCA037-17	MG191659
L. erinaceum	8	NCCA0133a	NOCCA040-17	MG191678
L. erinaceum	8	NCCA0135a	NOCCA043-17	MG191683
L. erinaceum	2A	NCCA0137	NOCCA045-17	MG191680<br>MG191373
L. erinaceum	9	NCCA0144	NOCCA050-17	MG191679
L. erinaceum	9	NCCA0145	NOCCA051-17	MG191702
L. erinaceum	12	NCCA0150	NOCCA056-17	MG191677
L. erinaceum	15	NCCA0155	NOCCA060-17	MG191661
L. erinaceum	19	NCCA0159	NOCCA064-17	MG191671
L. erinaceum	21	NCCA0172	NOCCA073-17	MG191700
Lithothamnion cf. *glaciale* Kjellman	8	NCCA0132a	NOCCA082-17	MG191673
L. cf. *glaciale*	8	NCCA0134b	NOCCA091-18	MH034113
L. cf. *glaciale*	2A	NCCA0138	NOCCA046-17	MG191696
L. cf. *glaciale*	9	NCCA0146	NOCCA052-17	MG191660
L. cf. *glaciale*	12	NCCA0147	NOCCA053-17	MG191687
L. cf. *glaciale*	12	NCCA0148	NOCCA054-17	MG191688
L. cf. *glaciale*	12	NCCA0149	NOCCA055-17	MG191690
L. cf. *glaciale*	12	NCCA0151	NOCCA083-17	MG191692
L. cf. *glaciale*	15	NCCA0152	NOCCA057-17	MG191699
L. cf. *glaciale*	15	NCCA0153	NOCCA058-17	MG191698
L. cf. *glaciale*	15	NCCA0154	NOCCA059-17	MG191681

Taxonomy	Dive site	Project ID	Bold ID	Acc. Nr.
L. cf. *glaciale*	15	NCCA0156	NOCCA061-17	MG191675
L. cf. *glaciale*	19	NCCA0157a	NOCCA062-17	MG191686
L. cf. *glaciale*	19	NCCA0162	NOCCA086-17	MG191685
L. cf. *glaciale*	19	NCCA0163	NOCCA087-17	MG191693
L. cf. *glaciale*	19	NCCA0164	NOCCA066-17	MG191695
L. cf. *glaciale*	20	NCCA0165	NOCCA067-17	MG191667
L. cf. *glaciale*	20	NCCA0166	NOCCA068-17	MG191663
L. cf. *glaciale*	20	NCCA0167	NOCCA069-17	MG191689
L. cf. *glaciale*	20	NCCA0168	NOCCA070-17	MG191674
L. cf. *glaciale*	20	NCCA0169	NOCCA071-17	MG191665
Phymatolithon borealis W.H. Adey, J.J. Hernandez-Kantun & P.W. Gabrielson	9	NCCA0142	NOCCA048-17	MG191694
P. borealis	9	NCCA0143	NOCCA049-17	MG191662
P. borealis	19	NCCA0157b	NOCCA063-17	MG191669
P. borealis	19	NCCA0158	NOCCA084-17	MG191701<br>MG191375
P. borealis	19	NCCA0160	NOCCA085-17	MG191668
P. borealis	19	NCCA0161	NOCCA065-17	MG191682
Phymatolithon calcareum (Pallas) W.H. Adey & McKibbin	21	NCCA0173	NOCCA088-17	MG191697
P. calcareum	21	NCCA0174	NOCCA089-17	MG191703
P. calcareum	21	NCCA0175	NOCCA090-17	MG191672
Phymatolithon cf. *rugulosum* W.H. Adey	8	NCCA0135b	NOCCA044-17	MG191666
P. cf. *rugulosum*	20	NCCA0170	NOCCA072-17	MG191658
Phymatolithon sp.	8	NCCA0132b	NOCCA039-17	MG191670
Phymatolithon sp.	8	NCCA0133b	NOCCA041-17	MG191664<br>MG191372
Phymatolithon sp.	8	NCCA0134	NOCCA042-17	MG191676

Figure S1. qPCR Ct and melt curve analysis results.

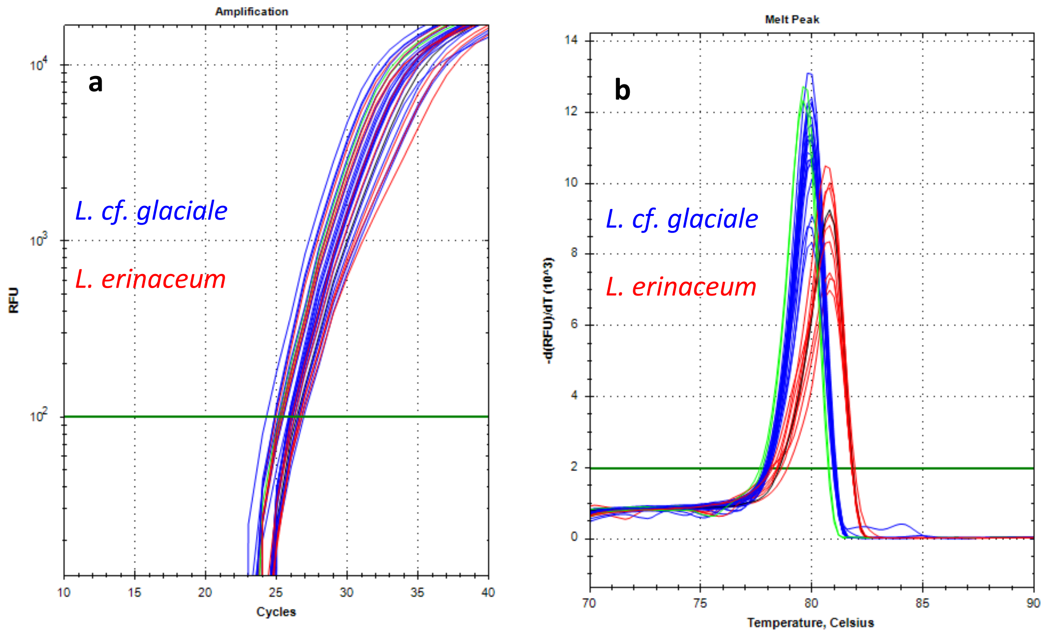

a & **b**) Blue shows all analyzed *Lithothamnion* cf. *glaciale*, red all analyzed *L. erinaceum* and green all *Phymatolithon* cf. *rugulosum* analyzed individuals. **a)** Ct results for all 32 samples used in the High Resolution Melt (HRM) analysis (see Figure 3). **b)** Melt curve analysis for all 32 samples used in the HRM analysis (see Figure 3).

Figure S2. Anatomical examination of sequenced specimens (SEM).

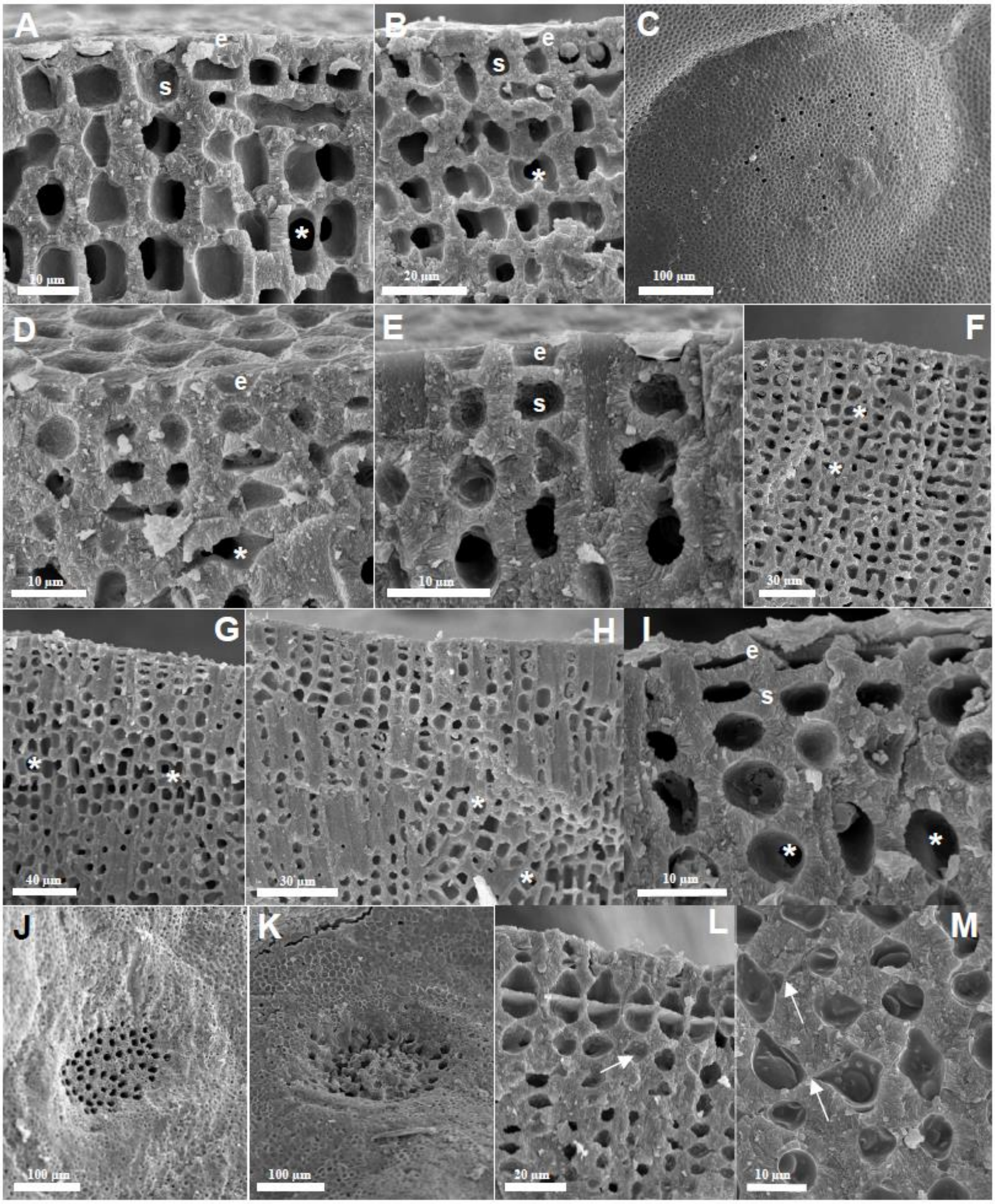

A-C) *Lithothamnion erinaceum.* A-B: flared epithallial cells (e), subepithallial cells (s) longer than cells subtending them, and cell fusions (*) between cells of contiguous filaments; C) surface view of sporangial conceptacles multiporate, raised and without rim. D-F) *Lithothamnion* cf. *glaciale*. Vertical sections showing flared epithallial cells (e) subepithallial cells (s) longer than cells subtending them, and cell fusions (*) between cells of contiguous filaments. G-K) *Phymatolithon* spp. G: Vertical

section of *P. calcareum* showing c cell fusions (*) between cells of contiguous filaments. H: Vertical section of *P.* cf. *rugulosum* showing c cell fusions (*) between cells of contiguous filaments. I: Vertical section of *P. borealis* showing domed epithallial cells (e) subepithallial cells (s) shorter than cells subtending them, and cell fusions (*) between cells of contiguous filaments. J-K: multiporate sporangial conceptacles flushed with surface in *P. borealis*. L-M) *Lithophyllum* sp. Vertical section showing secondary pit-connections (arrows) between cells of contiguous filaments. Specimens examined: A, C: NCCA155; B: NCCA135A; D: NCCA152; E: NCCA132A; F: NCCA147; G: NCCA174; H: NCCA170; I: NCCA142; J: NCCA160; K: NCCA142; L-M: NCCA131B.

Figure S3. Full length gel for Fig. 1.

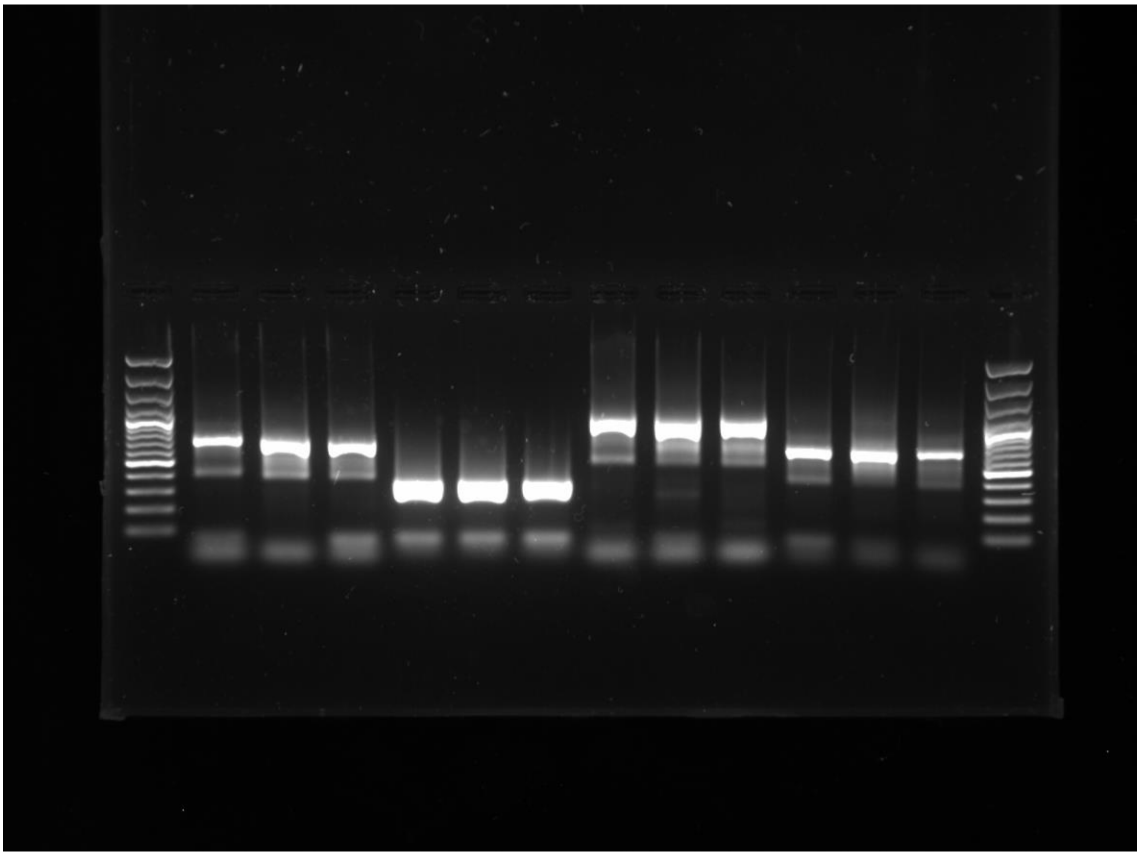

Supplement: Supplementary file 1 — Dataset 1 [file 41598_2018_36998_MOESM1_ESM.pdf]
